# Intestinal Spirochetosis: An Obscure Cause of Lower Gastrointestinal Bleeding

**DOI:** 10.7759/cureus.2970

**Published:** 2018-07-12

**Authors:** Kevin R Green, Ciel Harris, Asim Shuja, Miguel Malespin, Silvio W De Melo

**Affiliations:** 1 Internal Medicine, University of Florida College of Medicine-Jacksonville, Jacksonville, USA; 2 Division of Gastroenterology, University of Florida College of Medicine-Jacksonville, Jacksonville, USA; 3 Division of Gastroenterology, Oregon Health and Science University, Portland, USA

**Keywords:** intestinal spirochetosis, hematochezia, endoscopy, colitis

## Abstract

Adherence of spirochetes to the apical membrane of the colonic epithelium has been well-described in the literature, but the exact pathogenesis leading to symptomatic clinical manifestations is poorly understood. Most cases are found incidentally on the pathological evaluation of colonic biopsies taken during diagnostic or therapeutic colonoscopies. However, whether the colonization of the intestinal mucosa can be attributed to clinical symptoms is a matter of debate. Here, we present a case of intermittent hematochezia attributed to the overwhelming invasion of the colonic mucosa by intestinal spirochetes.

## Introduction

Spirochete belongs to the phylum Spirochaetes, which are thin, highly motile, gram-negative, double-membrane bacteria in which most species characteristically contain long spiral-shaped cells. The family is divided into three families: Spirochaetaceae, Leptospiraceae, and Brachyspiraceae. Spirochetes are pathogenic in humans causing several diseases, such as Lyme disease, leptospirosis, and syphilis. The two members of the Brachyspiracea family, Brachyspira aalborgi and Brachyspira pilosicoli, are the species most commonly associated with intestinal colonization in humans [1]. The clinical presentation of intestinal spirochetosis (IS) may range from asymptomatic colonization to fulminant colitis [2-3]. Although severe IS has been reported primarily in immunocompromised individuals, herein, we present a case of invasive IS in a healthy patient.

## Case presentation

**Figure 1:**
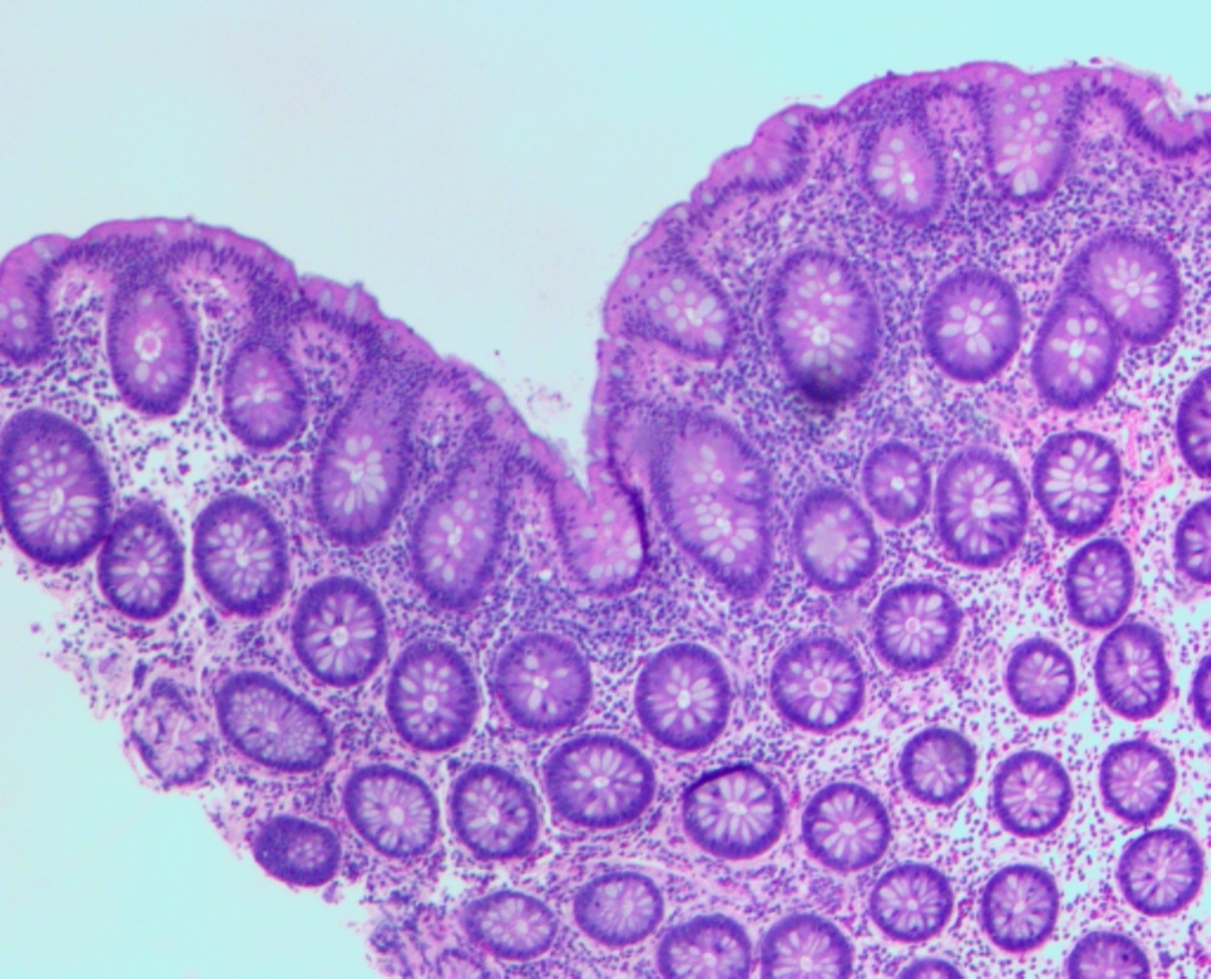
Terminal ileum hematoxylin-eosin stain

**Figure 2:**
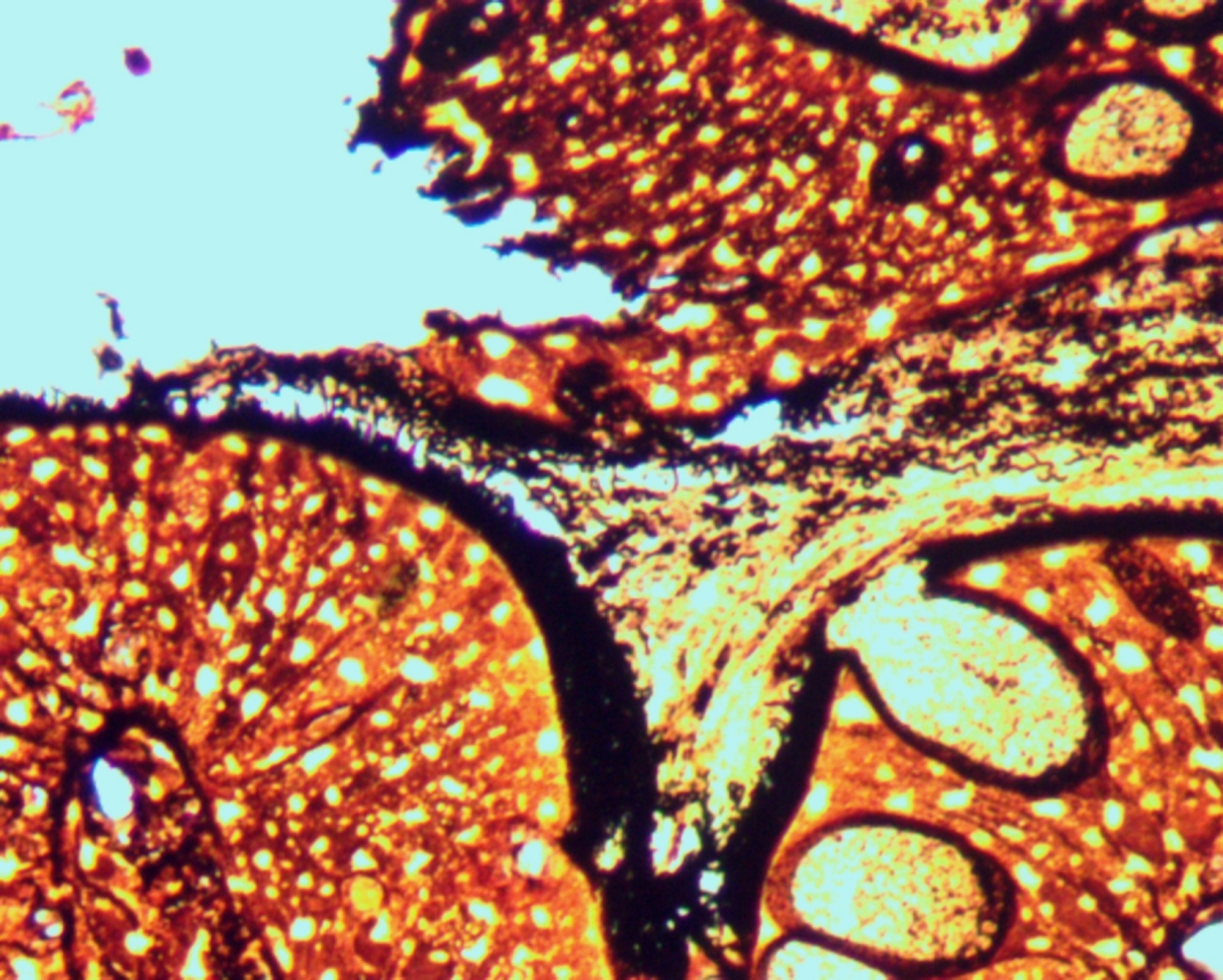
Warthrin-Starry stain of the terminal ileum

A 27-year-old male with no medical history presented to our emergency department with four days of atypical chest pain. He endorsed flu-like symptoms two weeks prior that failed to resolve with over-the-counter medications and amoxicillin. He also reported watery diarrhea and intermittent hematochezia. A complete cardiovascular workup was unremarkable. On admission, his hemoglobin decreased from 14 g/dl (baseline) to 10 g/dl due to a single episode of painless hematochezia. C-reactive protein was 6.5 and erythrocyte sedimentation rate was 60. All stool studies, including Clostridium difficile toxin, were negative. Coagulation studies were within normal limits. Physical exam was unremarkable, except for guaiac-positive stool. Colonoscopy revealed mild erythematous mucosa of the terminal ileum and a localized area of severely congested, erythematous, and inflamed mucosa in the rectum. Random biopsies were taken from the colon and rectum. IS were found in biopsies of the ascending colon, transverse colon, descending colon, sigmoid colon, rectum, and cecum. Terminal ileum and rectum biopsies also showed severe acute inflammatory changes with cryptitis and early crypt abscess formation (Figure 1). All histological samples displayed no evidence of inflammatory bowel disease. However, the presence of overwhelming spirochete invasion suggested these changes were secondary to IS (Figures 1-2). Testing for sexually transmitted diseases via polymerase chain reaction for human immunodeficiency, gonorrhea, chlamydia, herpes simplex virus, cytomegalovirus, and rapid plasma reagin was negative. He was treated with metronidazole and an improvement in symptoms was seen within three days (Kevin Green, Ciel Harris, Asim Suja, Miguel Malespin, Silvio de Melo, Jr.: Intestinal Spirochetosis: An Obscure Cause of Lower Gastrointestinal Bleeding--poster presentation. World Congress of Gastroenterology Mtg. October 17, 2017).

## Discussion

Intestinal spirochetosis (IS) was first described by Harland and Lee in 1967 as a condition in which anaerobic spirochetes adhere and colonize the luminal surface of colonic and appendiceal epithelium with occasional penetration into the epithelium [4]. The literature is rich in reports of incidentally discovered IS in asymptomatic patients. The data regarding severe symptomatology due to IS is scant; thus, the significance of IS remains a matter of debate [1, 5].

IS has been reported largely in developing countries; however, worldwide prevalence is unknown. One large study in the 1900s revealed a 28% prevalence among healthy individuals in Chicago [6]. A Norwegian study reported prevalence varying between 2.5% and 3% with a male predominance up to 75% [7]. Spirochetosis is estimated to affect up to 5% of heterosexuals and 30% of the homosexuals [3, 7]. The increase in prevalence among the homosexual population has been speculated to be due to increased oral-anal contact and anal intercourse [6]. Our patient endorsed being sexually active with multiple females and males but denied a history of sexually transmitted diseases or immunodeficiency.

Most cases of IS are asymptomatic, incidentally found on screening colonoscopies [8]. Symptomatic cases may present with chronic watery diarrhea and abdominal pain. Mild to moderate disease may be associated with hematochezia, while rare cases of severe disease have been associated with failure to thrive, fulminant colitis, and death [2]. The endoscopic appearance of the colon provides no value in the diagnosis of IS. Findings usually include non-specific hyperemic mucosa [7-8]. Due to the lack of hallmark symptoms or specific laboratory findings, the diagnosis is challenging.

Diagnosis of IS has traditionally been made by the histological appearance of a widespread blue fringe (on hematoxylin-eosin staining) 3 to 6 μm, along with the colonic epithelium, known as the “false brush border”, as seen in Figure 1 [4]. Histologic findings suggestive of IS may be followed by a Warthrin-Starry or Steiner silver impregnation stain to highlight the presence of spirochetes as seen in Figure 2. The presence of spirochetes attached end-on-end to the epithelial surface on transition electron microscopy serves as a confirmatory study [1-2, 8]. Interspecies variation of Brachyspira can be determined by molecular analysis using fluorescence in situ hybridization (FISH) and polymerase chain reaction (PCR) [9].

A retrospective study performed by Anthony et al. attempted to correlate the presence or absence of gastrointestinal symptoms with endoscopic or pathologic inflammation. The study reviewed 26 patients with biopsy-proven IS. Diarrhea or changes in bowel habits (46%) were the leading indications for endoscopy, followed by abdominal pain and rectal bleeding (31%). Only 19% of cases with spirochete colonization showed either endoscopic or pathologic inflammation [7].

Many cases of IS are asymptomatic and require no treatment. Antibiotic therapy is reserved for severely symptomatic cases that fail to resolve or cause functional limitations. No consensus exists in the literature with regards to dosing and duration of antibiotic therapy due to wide-ranging responses to treatment. Treatment results ranging from no significant improvement to complete resolution of clinical symptoms and normalization of colonic mucosae have been reported in the literature [1]. The degree of mucosal involvement has been proposed as a prognostic factor for response to therapy, but this currently is not evidence-based [8]. Clindamycin and macrolides have shown limited symptomatic improvement. Calderaro et al reported an 88% eradication rate using metronidazole which was confirmed via histological examination and PCR in 17 patients with IS [10]. Our patient was discharged home with metronidazole and reported improvement in symptoms within three days with no recurrence.

## Conclusions

This case demonstrates that invasive IS may develop in healthy individuals. Although many cases of asymptomatic IS have been well-described, cases leading to severe gastrointestinal symptoms are rare. Additionally, no current methods exist to prove a direct relationship between histologic findings of IS and active disease, thus leaving IS as the diagnosis of exclusion.
